# Observation of Strong Synergy in the Interfacial Water Response of Binary Ionic and Nonionic Surfactant Mixtures

**DOI:** 10.1021/acs.jpclett.2c02750

**Published:** 2022-12-01

**Authors:** Sanghamitra Sengupta, Rahul Gera, Colin Egan, Uriel N. Morzan, Jan Versluis, Ali Hassanali, Huib J. Bakker

**Affiliations:** † AMOLF, Science Park 104, 1098 XGAmsterdam, The Netherlands; ‡ Condensed Matter and Statistical Physics Centre, International Centre for Theoretical Physics, Strada Costiera 11, 34151Trieste, Italy

## Abstract

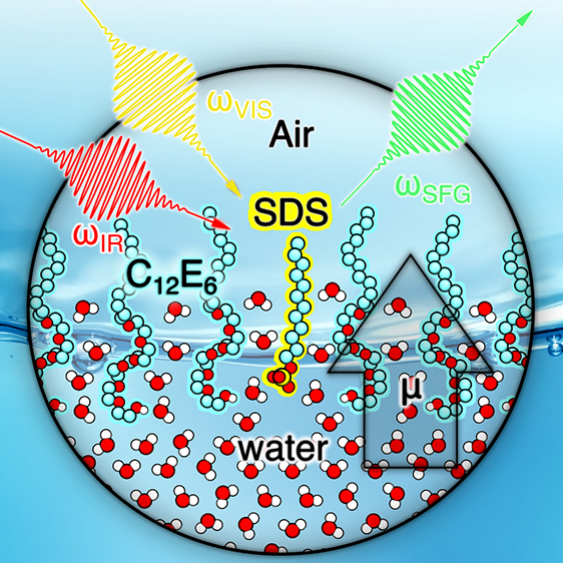

Interfacial vibrational footprints of the binary mixture of sodium dodecyl sulfate (SDS) and hexaethylene glycol monododecyl ether (C_12_E_6_) were probed using heterodyne detected vibrational sum frequency generation (HDVSFG). Our results show that in the presence of C_12_E_6_ at CMC (70 μM) the effect of SDS on the orientation of interfacial water molecules is enhanced 10 times compared to just pure surfactants. The experimental results contest the traditional Langmuir adsorption model predictions. This is also evidenced by our molecular dynamics simulations that show a remarkable restructuring and enhanced orientation of the interfacial water molecules upon DS^–^ adsorption to the C_12_E_6_ surface. The simulations show that the adsorption free energy of DS^–^ ions to a water surface covered with C_12_E_6_ is an enthalpy-driven process and more attractive by ∼10 *k*_B_*T* compared to the adsorption energy of DS^–^ to the surface of pure water.

Surfactants are a special class of amphiphilic organic molecules that have a high surface propensity in solutions of water and other polar liquids. Surfactants also often possess a unique capability of forming supramolecular assemblies among themselves^1,2^ and with various other molecular systems such as proteins^3^ and carbon nanotubes.^4,5^ The study of the molecular properties of these assemblies has emerged as an important research topic due to their enormous potential in biological,^6−9^ industrial,^10,11^ and environmental applications.^12^ Motivated by the widespread applications of surfactants, many experimental,^13−17^ and theoretical^18^ studies have been devoted to the understanding of intermolecular surfactant interactions, both in the bulk and at interfaces. Despite extensive research focusing on such interactions, there is still a myriad of open questions, mostly because such interactions are molecule-specific and vary greatly with the size and nature of the hydrophilic and hydrophobic parts of the surfactants under investigation.^19^

Recent studies showed that solutions containing mixtures of different types of surfactants possess highly interesting properties. For instance, mixtures of anionic and nonionic surfactants were found to play a crucial role in protein folding–refolding processes^20−22^ and in determining protein conformation.^23^ It has been shown that nonionic surfactants form mixed micelles with ionic surfactants and reduce the interaction between the ionic surfactant and the protein under study.^21^ Mixed surfactant systems are also deployed for separating single-walled carbon nanotubes^24^ and the dissolution of membrane proteins.^25^ Mixing of neutral and ionic surfactants provides a manner in which to tune the protein folding–unfolding process.^26^ However, a molecular-level understanding of the underlying mechanism is lacking. Up to now, very few studies have been devoted to binary mixtures of surfactants. These studies have been limited to solutions of surfactants at their respective Critical Micellar Concentrations (CMC) or macroscopic studies of the properties of these solutions.^27^ As a result, the molecular-scale structure of the surface of these systems is still poorly understood. Obtaining this understanding can be extremely beneficial to address surfactant-driven and surface-mediated biophysical processes.

In this manuscript, we report on the interfacial structure of binary solutions of sodium dodecyl sulfate (SDS) and hexaethylene glycol monododecyl ether (C_12_E_6_) at different bulk concentration ratios using heterodyne detected vibrational sum-frequency generation spectroscopy (HDVSFG).^28^ Both SDS and C_12_E_6_ are widely used surfactant systems and have been studied extensively before. The interfacial structure of aqueous solutions of C_12_E_6_ has recently been studied using HDVSFG, Kelvin-probe measurements, and molecular dynamics (MD) simulations.^29^ In this study, it was found that C_12_E_6_ at CMC (70 μm) generates a strong electric field of 1 V/nm arising from the orientational structure of both the hydrophobic and hydrophilic parts of the surfactant and water molecules. The SDS–water interface has also been probed previously using spectroscopic techniques over a broad frequency range.^30−32^ These studies showed that the negative charge of the amphiphilic DS^–^ ion induces an extended orientation of the water molecules in the vicinity of the water surface. However, how the mixed surfactant systems alter the surface electric field remains quite an unexplored topic. In this work, we investigated the resultant effect of SDS and C_12_E_6_ binary system at the water interface.

We find that SDS and C_12_E_6_ have a strong synergistic effect on the structure of the near-surface water layers and that the properties of binary aqueous solutions of ionic and nonionic surfactants cannot be described with a conventional Langmuir adsorption model.^33,34^ A detailed discussion of the Langmuir adsorption model for both single solute systems and binary mixtures is discussed in SI 1. We rationalize the synergy of SDS and C_12_E_6_ using molecular dynamics simulations that show that the presence of C_12_E_6_ at the water surface serves as an attractive sink for DS^–^ ions and that the uptake of DS^–^ in the C_12_E_6_ layer is accompanied by large changes in the hydrogen-bond network of the water layers near the surface.

In Figure 1 we show HDVSFG spectra in the frequency range between 2750 and 3675 cm^–1^ of an aqueous solution containing 700 nM SDS, an aqueous solution containing 70 μM C_12_E_6_, and an aqueous solution containing both 700 nM SDS and 70 μM C_12_E_6_. Detailed information regarding the sample source and preparation used in this manuscript and the HDVSFG spectrometer can be found in SI 8. We present the imaginary component of the second-order susceptibility Im[χ^(2)^] measured for these solutions. The HDVSFG spectrum of water shows a weak broad negative band between 3200 and 3600 cm^–1^, whereas the HDVSFG spectra of the solutions of SDS and C_12_E_6_ show two strong positive bands (3240 and 3400 cm^–1^). These responses are all attributed to the O–H stretch vibrations of different hydrogen-bonded water molecules.^35,36^ The negative sign of the broad OH signal observed for pure water indicates that the hydrogen-bonded OH groups of the water molecules have a net orientation toward the bulk. The positive sign of the OH signals observed for the surfactant solutions shows that the hydrogen-bonded OH groups have a net orientation away from the bulk, which for SDS can be well explained by the negative charge of the dodecyl sulfate (DS^–^) ions accumulated at the surface.^30,31^ For C_12_E_6,_ the net orientation of the water OH groups toward the surface results from the formation of hydrogen bonds to the ether oxygen of the headgroup of the surfactant.^29^

**Figure 1:**
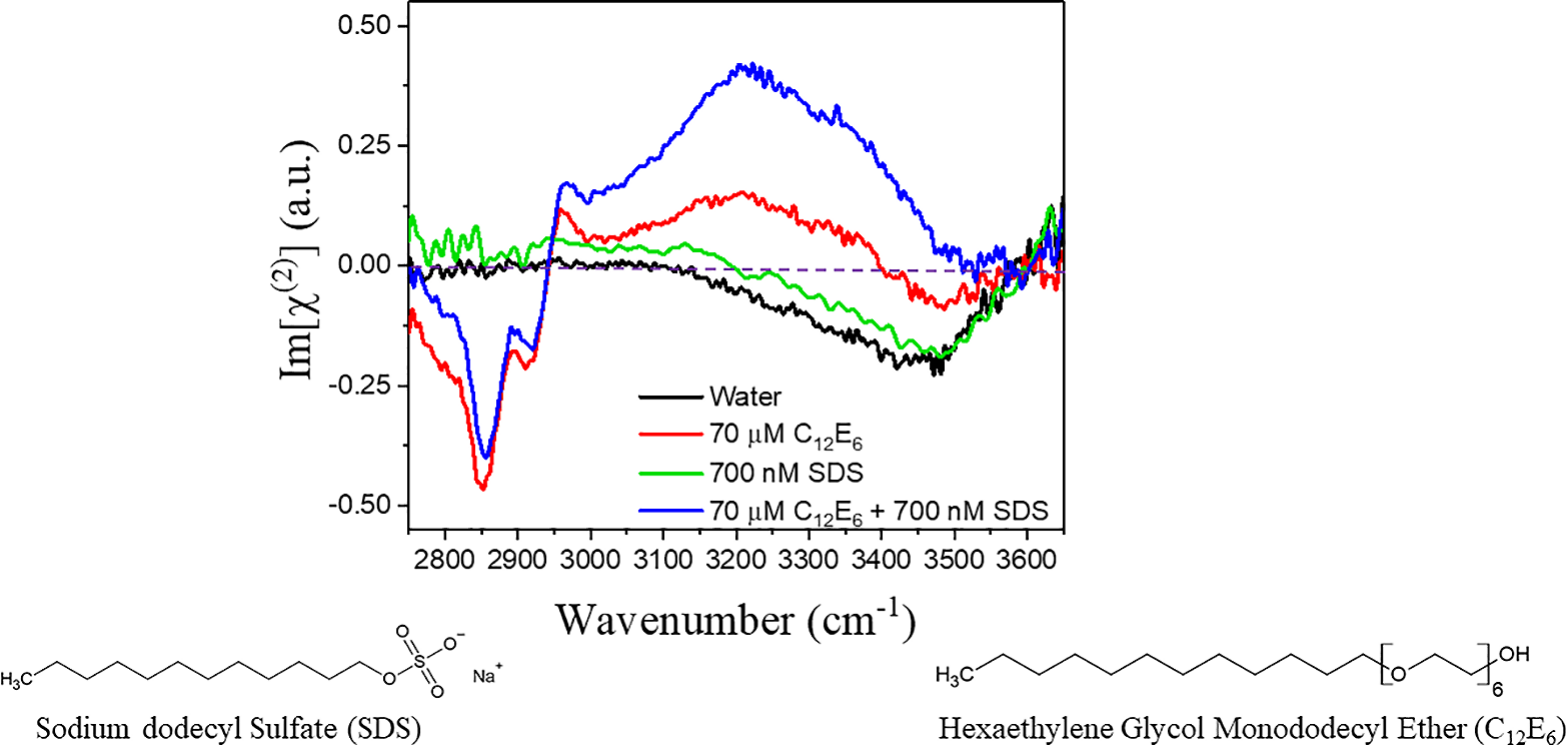
Heterodyne detected vibrational sum-frequency generation (HDVSFG) spectra (imaginary χ^(2)^) of pure water (black), an aqueous solution of 70 μm C_12_E_6_ (red), an aqueous solution of 700 nM SDS (green), and an aqueous solution containing both 700 nM SDS and 70 μm C_12_E_6_ (blue). The molecular structures of SDS (left) and C_12_E_6_ (right) are shown below the spectra.

The HDVSFG spectra of the solutions containing C_12_E_6_ show two strong negative features at 2850 and 2920 cm^–1^ and a small positive band at 2965 cm^–1^. Following earlier work on systems analogous to C_12_E_6_ by the group of Tyrode,^37^ we assign the band at 2850 cm^–1^ to the symmetric C–H stretch vibrations of the methylene (CH_2_) groups and the terminal CH_3_ group of the aliphatic chain of C_12_E_6_, with a dominant contribution of the CH_2_ groups. The negative band at 2920 cm^–1^ is assigned to the Fermi resonance of the symmetric C–H stretch vibrations and the overtones of the C–H bending mode of the CH_2_ and CH_3_ groups.^30,38^ The small positive band at 2965 cm^–1^ is assigned to the antisymmetric C–H stretch vibration of the terminal CH_3_ group. The negative sign of the symmetric stretch vibrational bands and the positive sign of the asymmetric stretch vibrational band indicate that the aliphatic tails of the C_12_E_6_ surfactant molecules are pointing toward the air, away from the solution, as expected.

A surprising and exciting feature in Figure 1 is that the water signal of the solution containing both surfactants is much stronger than the added signal of the two solutions separately containing only C_12_E_6_ or SDS. The presence of C_12_E_6_ at CMC in the solution enhances the response of the water molecules to the addition of 700 nM SDS by a factor of ∼10.

In Figure 2 we show HDVSFG spectra of binary mixtures of SDS and C_12_E_6_ over a wide range of SDS concentrations. The HDVSFG spectra of SDS solutions only at the same concentrations as used in the binary mixture are reported in the Supporting Information (Figure 1 in SI 2). We observe a steady increase in the intensity of the water signal with increasing SDS concentration in both ranges of SDS concentrations where C_12_E_6_ is in excess (Figure 2a) or SDS is in excess (Figure 2b). To check whether the observations may be influenced by heat-induced effects, we repeated the measurement at one of the intermediate concentrations with a much reduced infrared power. This did not change the results (Figure 3 in SI 3). Additional surface tension measurements for these binary mixture conditions are also reported in SI 7. The responses of the C–H vibrations of the packed monolayer of surfactants remain constant with SDS concentration. The apparent change in the amplitudes of the C–H vibrations with SDS concentration is due to the rise of the low-frequency wing of the response of the water O–H vibrations with increasing SDS concentration. The positive water response of C_12_E_6_ just by itself is explained in detail elsewhere.^29^ To briefly mention, the positive water signal between 3000 and 3400 cm^–1^ for solutions of C_12_E_6_ results from the strong hydrogen bonding of water molecules to the ether groups of the headgroup of C_12_E_6_. The negative water signal around 3450 cm^–1^ observed for a solution containing only C_12_E_6_ has been attributed to weakly hydrogen-bonded water molecules located in between the hydrophobic tails of the surfactant molecules. For these water molecules, the O–H groups are oriented toward the bulk, thus yielding a negative HDVSFG signal. The addition of SDS generates an overall broad positive water signal due to the strong orientation effect of the negative charge on the water near the surface. This signal overshadows the negative water signal of the water molecules in between the hydrophobic tails of C_12_E_6_, leading to a net positive signal at all frequencies.

**Figure 2:**
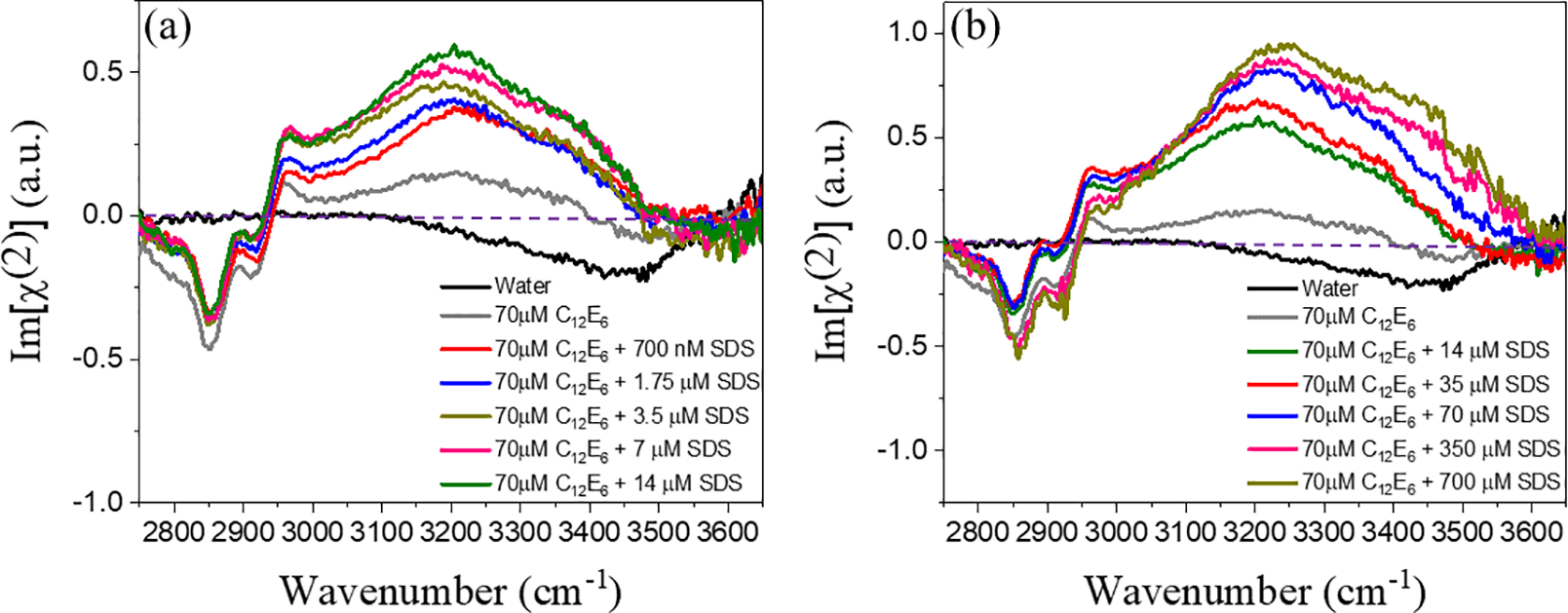
HDVSFG data of binary mixtures of C_12_E_6_ at CMC (70 μM) and different concentrations of SDS. In the left panel, the ratio between C_12_E_6_ and SDS varies from 100:1 to 5:1, and in the right panel, the ratio varies from 5:1 to 1:10. For clarity, the spectrum of the solutions with a ratio of C_12_E_6_ to SDS of 5:1 is shown in both panels using the same color. We observe a steady increase in the water signal (between 3200 and 3600 cm^–1^) as we go up in SDS concentration in the HDVSFG spectra.

To study the synergetic effect of SDS and C_12_E_6_ quantitatively, we show in Figure 3 the ratio of the enhanced water signal ranging between 3200 and 3600 cm^–1^ induced by SDS both in the presence of 70 μm C_12_E_6_ and in the absence of C_12_E_6,_ as a function of the concentration SDS. The synergistic effect was calculated using the following equation , where “S” indicates the maximum amplitude of the water signal for different solutions. A ratio larger than 1 implies that there is a synergetic effect on the water signal. It is seen that the synergetic effect of SDS and C_12_E_6_ on the water signal is very strong for SDS concentration up to ∼3.5 μM SDS (Figure 3 inset) and vanishes (the ratio attaining a value of ∼1) at an SDS concentration of ∼70 μM. At SDS concentrations >70 μM the ratio drops below 1, showing that at these concentrations the competition of the two surfactants for the limited surface area becomes more important than the synergetic effect.

**Figure 3:**
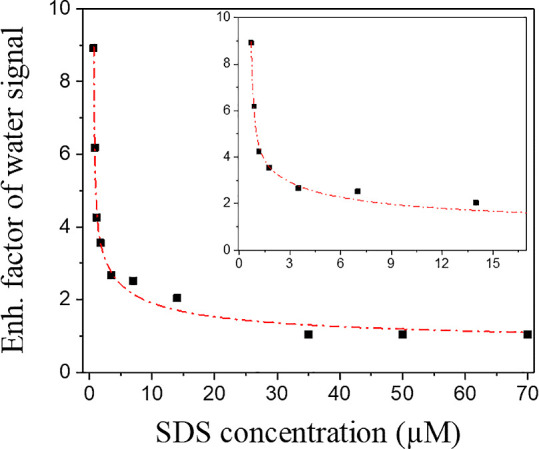
Magnitude of the synergistic effect of SDS and C_12_E_6_ on the response of the water O–H stretch vibrations as a function of the concentration SDS. The amplitude of the synergistic effect was calculated as the ratio of the water signal induced by SDS in the presence of 70 μm C_12_E_6_ and the absence of C_12_E_6_, as a function of the concentration SDS, which can mathematically be expressed as . The squares markers are the experimental values, and the dotted line is a guide to the eye. The inset shows a zoom-in of the concentration range 700 nM to 15 μM SDS.

We performed molecular dynamics simulations to identify the driving force behind the synergy of SDS and C_12_E_6_ on the response of the interfacial water molecules. An elaborate discussion of the details of the computation methods and packages used in this research work is described in SI 8. We employed the umbrella sampling (US) technique (see Computational Methods in SI 8) to compare the binding free energy due to DS^–^ adsorbing to the bare air–water interface with that of DS^–^ adsorbing to a surface fully covered with C_12_E_6_. Panels a, b, and c of Figure 4 visually depict the three systems that are simulated, i.e., DS^–^ adsorbing to the water–air interface, the water–C_12_E_6_–air interface (without DS^–^), and DS^–^ adsorbing to the water–C_12_E_6_–air interface, respectively. From our simulations, we find that, at low concentrations, interfacial DS^–^ tends to orient nearly parallel to the surface when adsorbed to the water–air interface and nearly perpendicular to the water surface when adsorbed to the water–C_12_E_6_–air interface, as depicted in panels a and c, respectively.

**Figure 4:**
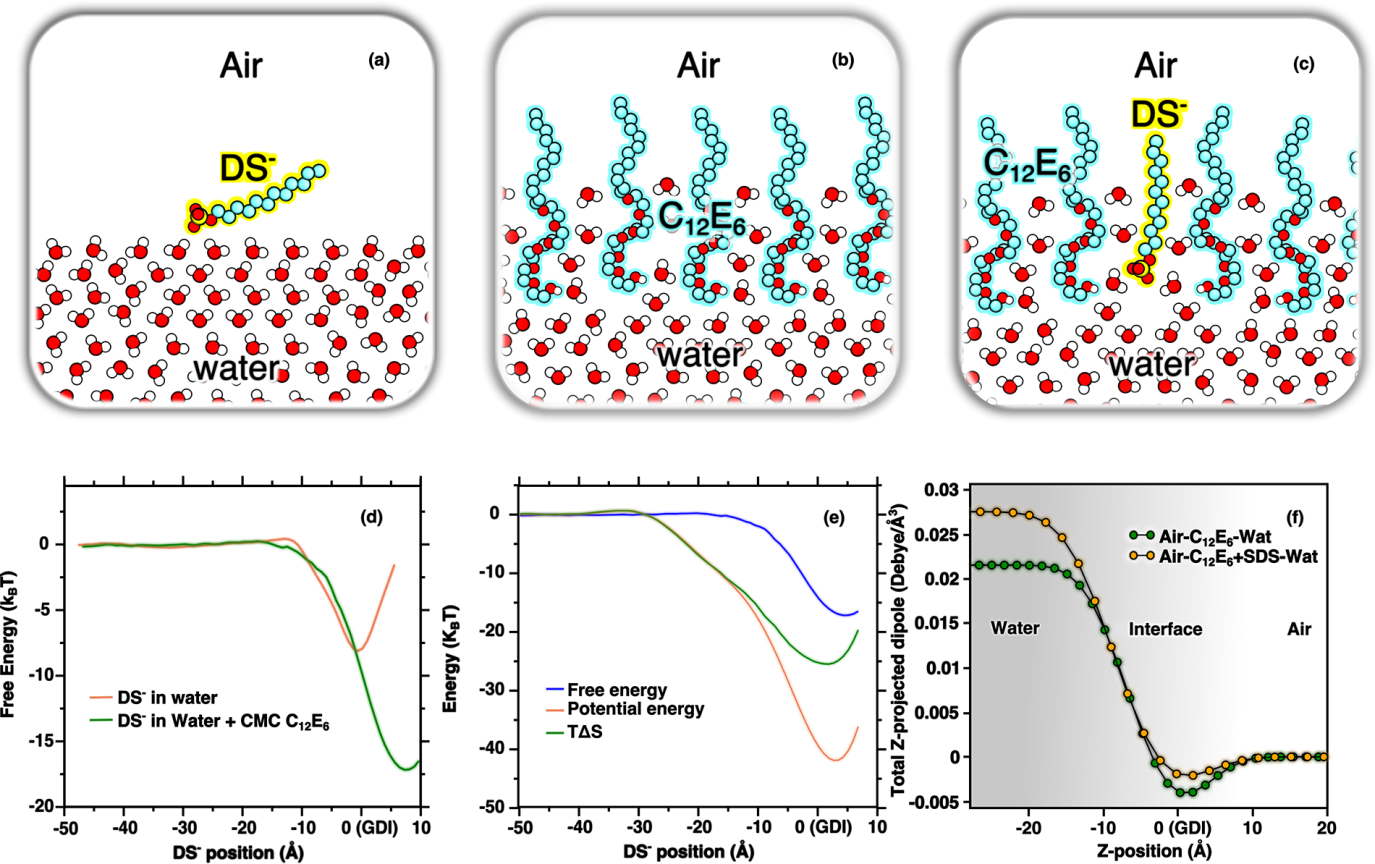
Panels (a), (b), and (c) depict the three systems simulated. Panel (d) shows the free energy (*k*_B_*T*) associated with DS^–^ adsorbing to the bare air–water interface (orange curve) and DS^–^ adsorbing to the water–C_12_E_6_–air interface at CMC surface coverage (green curve), as a function of the DS^–^ position (in angstroms) relative to the GDI of the water phase. The curves are computed with the umbrella sampling method. Panel (e) shows the decomposition of the free energy of DS^–^ adsorbing the water–C_12_E_6_–air interface (blue curve), into enthalpic and entropic contributions (orange and green curves, respectively) as a function of the DS^–^ position. Panel (f) depicts the integrated dipole density per unit volume in the perpendicular direction to the water interface (*Z*-axis).

Figure 4d shows a comparison between the free energy profiles computed for each system as a function of the DS^–^ distance from the interface. Zero distance corresponds to the Gibbs dividing interface where the water density has half its bulk value. We find that the binding free energy of DS^–^ adsorbing to a bare water–air interface is −8 *k*_B_*T*, while that of DS^–^ adsorbing to the water–C_12_E_6_–air interface is −19 *k*_B_*T*. This roughly 10 *k*_B_*T* enhancement of the binding free energy due to the presence of a C_12_E_6_ monolayer at the interface leads to a much higher surface concentration of DS^–^ for a water surface that is covered with a layer of C_12_E_6_ than for the bare water surface.

The simulations allow for the determination of the entropic and enthalpic contributions of DS^–^ binding to the water–C_12_E_6_–air interface, as shown in Figure 4e (the decomposition of the entropic and enthalpic contributions of DS^–^ binding to the air–water interface is shown in SI 5). The results show that the enhancement of the free energy for the adsorption of DS^–^ is largely enthalpic. The binding of DS^–^ to the surface is favored enthalpically by roughly −45 *k*_B_*T* while the entropic term incurs a penalty of approximately 25 *k*_B_*T*. From our simulations, we find that the large enthalpic stabilization is driven by a combination of van der Waals packing interactions between the hydrophobic chains (∼80%) and electrostatic interactions of the ether groups of the headgroup of C_12_E_6_ with the headgroup of DS (∼20%).

The observation that the binding of DS^–^ to the water–C_12_E_6_–air interface is enthalpically driven is somewhat surprising, as hydrophobic aggregation or self-assembly of aliphatic tails in aqueous environments is usually driven by a gain in entropy.^39,40^ In fact, for DS^–^ binding to a bare water surface (no C_12_E_6_), the adsorption is entropically driven (SI 5). The fact that the adsorption of DS^–^ to a C_12_E_6_-covered water surface is enthalpy-driven can be explained as follows. First, there is a drastic change in DS^–^ orientation leading to favorable van der Waals interactions between the aliphatic hydrocarbon chains (Figure 4c). Furthermore, previous work showed that C_12_E_6_ creates a 3 nm thick polarized layer of water at the interface^29^ which is not present in pure water. When DS^–^ absorbs at the water–C_12_E_6_–air interface, this polarized water layer gets even thicker. This extended polarized water layer constitutes a significant attractive enthalpic contribution (SI 5). In addition, besides the role of the water, the simulations also suggest important contributions coming from changes in both the sodium counterion and C_12_E_6_ upon DS^–^ binding to the surface. The extended orientation of the water molecules at the water–C_12_E_6_–air interface limits their conformational space, which largely explains the entropic penalty of approximately 25 *k*_B_*T*, associated with the adsorption of DS^–^ to the interface.

In Figure 4f the enhanced orientation of the water molecules induced by the adsorption of DS^–^ to the water–C_12_E_6_–air interface is illustrated by calculating the integrated water dipole densities per unit volume as a function of the perpendicular direction to the interface (Z-coordinate). Note that the dipole is integrated from the air toward the bulk. The net dipole caused by the orientation of water clearly shows a very strong sensitivity to the presence of DS^–^. In agreement with the HDVSFG results illustrated in Figures 1 and 2, Figure 4f shows that adding SDS to the C_12_E_6_ at CMC leads to a significant enhancement of the orientation of the water molecules.

The present results demonstrate that the interaction between C_12_E_6_ and a negatively charged surfactant can be highly favorable and can have a profound effect on the structure and orientation of nearby water molecules. In recent years the study of the interaction of C_12_E_6_ with other organic and inorganic molecules has emerged as an important research topic due to its wide range of applications.^41^ For instance, it has been shown that nonionic surfactants form mixed micelles with ionic surfactants and reduce the interaction between the ionic surfactant and the protein under consideration.^21^ It has thus been found that the addition of a neutral surfactant to an SDS–protein complex reduces the protein denaturing capability of SDS and promotes the refolding of different membrane proteins from the SDS-bound complexes.^42−44^ The presently observed highly favorable interaction between C_12_E_6_ and SDS offers a potential explanation for this effect. Protein denaturation by SDS likely relies on the favorable interaction of the hydrophobic tail of the DS^–^ ion and the hydrophobic residues of the protein. When adding a neutral surfactant like C_12_E_6_ to complexes of DS^–^ and unfolded proteins, the favorable interaction between the hydrophobic tails of the DS^–^ ions and the hydrophobic residues will likely be replaced by the even more favorable interaction between the hydrophobic tails of C_12_E_6_ and SDS. As a result, the hydrophobic protein residues may detach from DS^–^ and reaggregate, implying a (partial) refolding of the protein.

In conclusion, we investigated the molecular properties of the surface of binary aqueous solutions SDS and C_12_E_6_ with surface-specific heterodyne detected vibrational sum-frequency generation (HDVSFG) and molecular dynamics simulations. In the experiments, we varied the SDS concentration from 700 nM to 700 μM while keeping the C_12_E_6_ concentration at CMC (70 μM). We observe that the orientation of water molecules at the surface resulting from the addition of SDS is enhanced by a factor of ∼10 in case the solution also contains C_12_E_6_ at CMC. The magnitude of this enhancement decreases when the SDS concentration is increased. According to the Langmuir model isotherm (mathematical formula given in SI 1), surfactants will compete for the available surface area. As a result, the surface occupancy of a surfactant will always become lower when another surfactant is added to the solution. As the surface signal of a particular surfactant is proportional to its surface occupancy, the signal collected from the surface of a mixture should be lower than the sum of the signals coming from the surfaces of solutions containing the separate components. Here we observe the opposite. The HDVSFG signal from the binary mixture is strongly enhanced compared to the individual components’ surface signals, which shows a clear violation of the Langmuir adsorption isotherm model. The origin of this synergetic effect is investigated with molecular dynamics simulations. These simulations show that the presence of C_12_E_6_ at the water surface enhances the free energy change associated with the adsorption of DS^–^ to the surface. The simulations also show that the enhancement of the free energy change is of enthalpic nature, which can be explained by the favorable interaction between DS^–^ and C_12_E_6_ at the surface and the collectively induced reorganization and enhanced orientation of the near-surface water layers.

The current study shows that the interaction between C_12_E_6_ and a negatively charged surfactant can be highly favorable and can strongly affect the structure of nearby molecules, including the hydrogen-bond structure and net dipolar orientation of nearby water layers. The interaction of differently charged surfactants is thus highly relevant for many systems, including membranes and protein–surfactant complexes. We hope that the present results will inspire future theoretical and experimental investigations.

## Supporting Information Available

Description of the Langmuir adsorption model for the binary mixture, HDVSFG spectra of pure SDS on bare water, effects of heating on interfacial structural degradation, comparison of decompositions of free energy profiles into enthalpic and entropic components for both systems simulated, energy decomposition of DS^–^ adsorption to the air–C_12_E_6_–water interface vs the bare air–water interface, raw average potential energy data illustrating the averaging method, surface tension measurements of the binary mixture, materials and methods (PDF)
Transparent Peer Review report available (PDF)
