# Supplementary material for: Observation of Strong Synergy in the Interfacial Water Response of Binary Ionic and Nonionic Surfactant Mixtures
Source: J Phys Chem Lett. 2022 Dec 1;13(49):11391–7. doi: 10.1021/acs.jpclett.2c02750 (PMC9761666; doi:10.1021/acs.jpclett.2c02750)
Supplement: Supplementary file 1 — jz2c02750_si_001.pdf [file jz2c02750_si_001.pdf]

Observation of Strong Synergy in the Interfacial Water Response of Binary Ionic and Non-ionic Surfactant Mixtures

Sanghamitra Sengupta[1]†, Rahul Gera[1]†, Colin Egan[2]†, Uriel N. Morzan[2], Jan Versluis[1], Ali Hassanali[2], Huib J. Bakker[1].

[1]*AMOLF, Science Park 104, 1098 XG, Amsterdam, Netherlands*

[2]*Condensed Matter and Statistical Physics Centre, International Centre for Theoretical Physics*, Strada Costiera 11, 34151, *Trieste, Italy.*

Corresponding author's email: s.sengupta@amolf.nl

*†***The authors have contributed equally to the work.**

Supplementary Information

1. **Description of Langmuir adsorption model for the binary mixture.**
2. **HDVSFG spectra of pure SDS on bare water.**
3. **Effects of heating on interfacial structural degradation.**
4. **Comparison of decompositions of free energy profiles into enthalpic and entropic components for both systems simulated.**
5. **Energy decomposition of DS^- adsorption to air-$C_{12}E_6$-water interface vs bare air-water interface.**
6. **Raw average potential energy data illustrating the averaging method.**
7. **Surface tension measurements of the binary mixture**
8. **Materials and Methods**

SI 1: Description of Langmuir adsorption model for the binary mixture.

Langmuir's adsorption model can explain the surface adsorption process for ideal solutions. For a single-component solution (in our case only SDS or only $C_{12}E_6$) the surface occupancy can be expressed as equation (1) (SDS is used here as the prototype),

$$\theta_{SDS} = \frac{a_{SDS}\, C_{SDS}}{1 + a_{SDS}C_{SDS}} \qquad (1)$$

Where the 'a'terms are the rate constants of the surface adsorption process and the 'C' terms denote the concentration of the respective surfactant molecules.

For the binary mixture, this model predicts that the constituent particles should compete for the same surface sites to adsorb/occupy. Under the assumption that particles are non-interacting, the surface is homogeneous, and a particular surface site can't be occupied by SDS and $C_{12}E_6$ simultaneously, we can express the surface occupancy/concentration (θ) of SDS and $C_{12}E_{6\,s}$ as follows,

$$\theta_{SDS} = \frac{a_{SDS}C_{SDS}}{1 + a_{C_{12}E_6}C_{C_{12}E_6} + a_{SDS}C_{SDS}} \qquad (2) \qquad \theta_{C_{12}E_6} = \frac{a_{C_{12}E_6}C_{C_{12}E_6}}{1 + a_{C_{12}E_6}C_{C_{12}E_6} + a_{SDS}C_{SDS}} \qquad (3)$$

So the relative ratio of the surface occupancy becomes,

$$\frac{\theta_{SDS}}{\theta_{C_{12}E_6}} = \frac{a_{SDS}C_{SDS}}{a_{C_{12}E_6}C_{C_{12}E_6}} \qquad (4)$$

Since the rate constants are fixed, the surface occupancy of each surfactant should be directly proportional to their respective bulk concentrations. This is also obvious from both equations (1) and (2) or (3), that the denominator is much larger in the latter case, resulting in smaller θ values in the case of for SDS and $C_{12}E_6$ in the mixture. The physical reasoning behind such a phenomenon is the intrinsic competition between the two participants. Hence for a binary mixture, we would expect in our HDVSFG spectra that Intensity (I) which is proportional to the surface occupancy of each of the constituents, $I_{SDS+\ C_{12}E_6} < I_{SDS} + I_{C_{12}E_6}$. However, in our experimentally observed data, we have seen that $I_{SDS+\ C_{12}E_6} > I_{SDS} + I_{C_{12}E_6}$ by a factor of 10 which is due to the synergistic effect, hence strongly contradicts the traditional Langmuir adsorption model.

SI 2: HDVSFG spectra of pure SDS solutions at the water interface

The HDVSFG spectra were collected from the interface from pure SDS solutions in water. The concentrations of the SDS solutions that are shown here are the same as those used in the main manuscript in figure 2. We observed a steady increase in the water signal with increasing SDS concentration from 700 nM (red) to 70 µM (yellow). No symmetric CH_3 symmetric stretch at 2850 cm^{-1}, fermi resonance at 2920 cm^{-1}, and asymmetric CH_3 band at 2960 cm^{-1} were observed indicating that in these concentrations of bulk SDS a packed monolayer doesn't form, unlike the binary mixture data. The data become quite noisy around 2800 cm^{-1} due to normalization effects. Also to make sure what is the limiting case from where we can start seeing C-H stretches in the case of the SDS solutions, we have collected HDVSFG data from 70 µM SDS and higher concentrations in this region as well (Figure 2). The data clearly shows that around 350 µM we start seeing the traces of C-H stretches between 2850 and 3100 cm^{-1} region and it only becomes prominent at around 700 µM bulk SDS concentration. This confirms that we do need at least

hundreds of micromolar of bulk SDS concentration for the C-Hs to appear or in other words a packed monolayer of SDS to be formed at the interface.

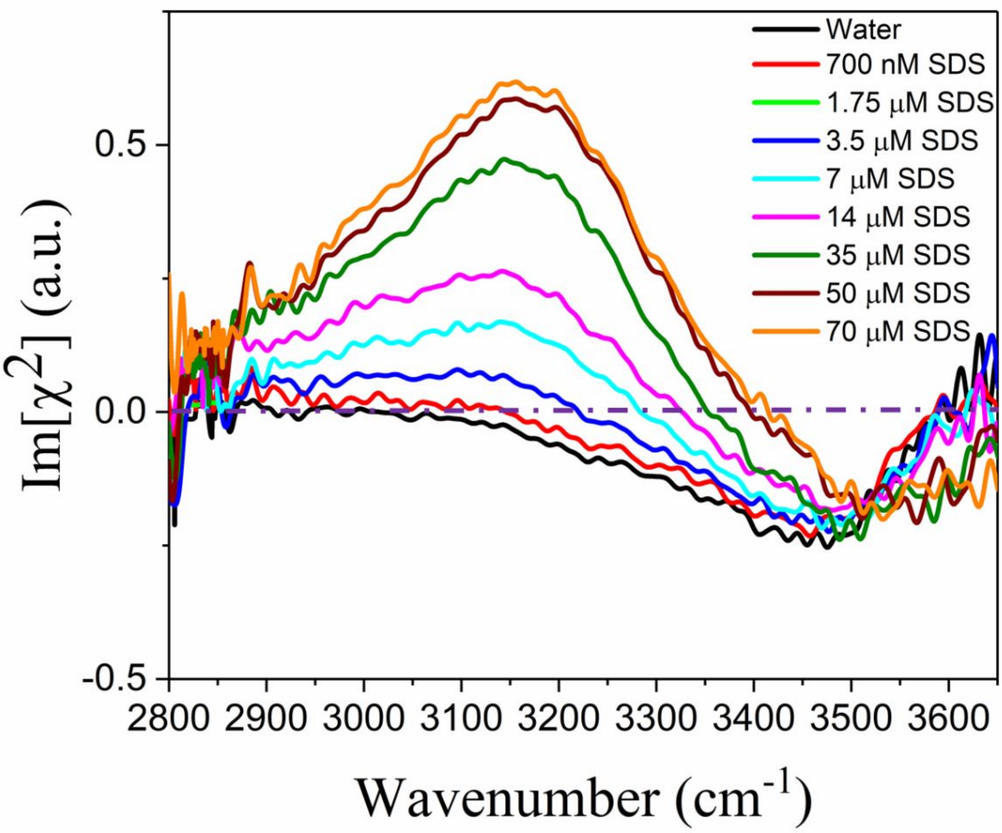

Figure 1: *Heterodyne detected spectra of pure SDS solutions of different concentrations varied from 700 nM to 70 μM. The spectra showed a steady increase in the water signal with increasing SDS concentration.*

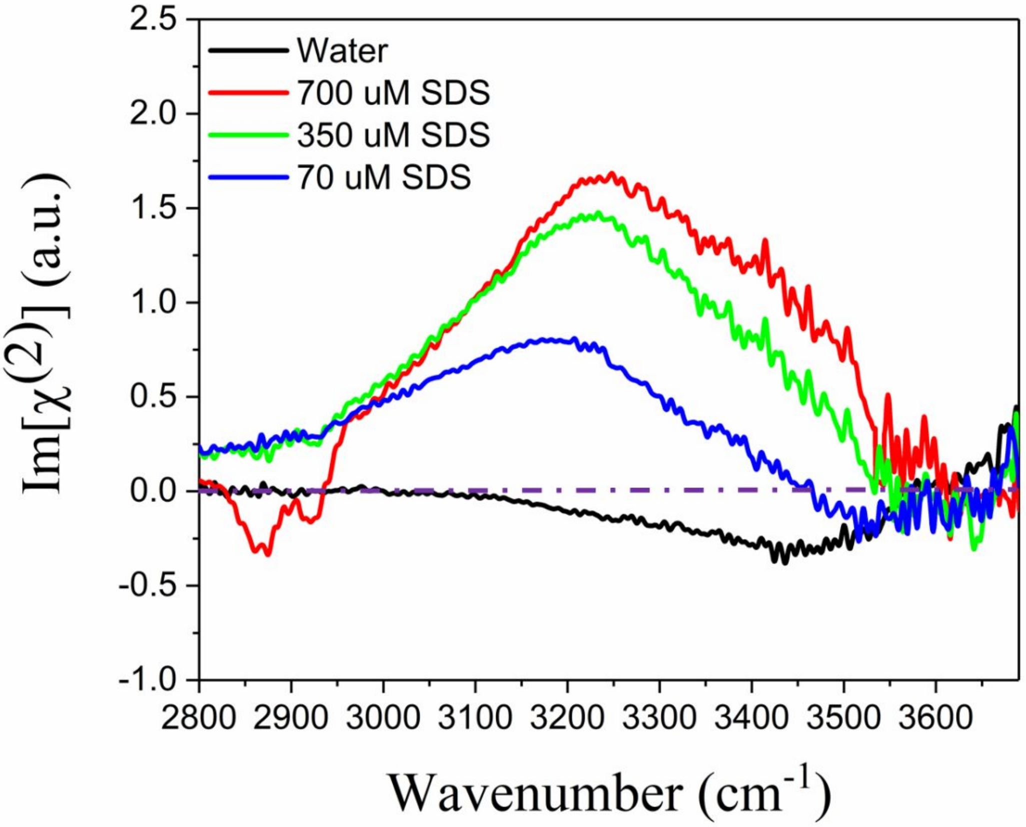

Figure 2: *This figure shows the comparison between three different concentrations of SDS (70 µM (blue), 350 µM (green), and 700 µM (red)). The C-H traces appear only at a much higher SDS concentration compared to 70 µM SDS and goes prominent at 700 µM, confirming that at < 70µM bulk SDS concentrations, there is no C-H stretches observed in HDVSFG spectra, and can only be observed at > hundreds of micromolar concentrations and hence a packed monolayer is not formed at those conditions.*

SI 3: Effects of heating on interfacial structural degradation

We have controlled the infrared (IR) power and reduced it to 5 mW from 8 mW to check that any heat-induced effects are observed on the interface of the binary mixture. Instead of an entire range of concentrations, we chose 70 µM SDS in presence of 70 µM $C_{12}E_6$ and compared the result with 70 µM $C_{12}E_{6.}$ The concentration of 70 µM of SDS was chosen due to a significant increase in water signal compared to the 70 µM $C_{12}E_6$. Also, compared to the lower SDS concentrations, at this concentration more SDS molecules are expected to be at the surface and hence more prone to show any heat-induced effect if any. Each sample data are an average of three individual spectrums acquired for 90 seconds each. We haven't observed any change in spectral features or within the concentration series, which indeed proves that there is no heat-induced effect for the binary mixture between the 5 mW to 8 mW IR pulse power range. Additionally, the ~ 3 times increase in the water signal between 70 µM $C_{12}E_6$ with and without 70 µM SDS remained the same for both 5 mW and 8 mW experimental conditions.

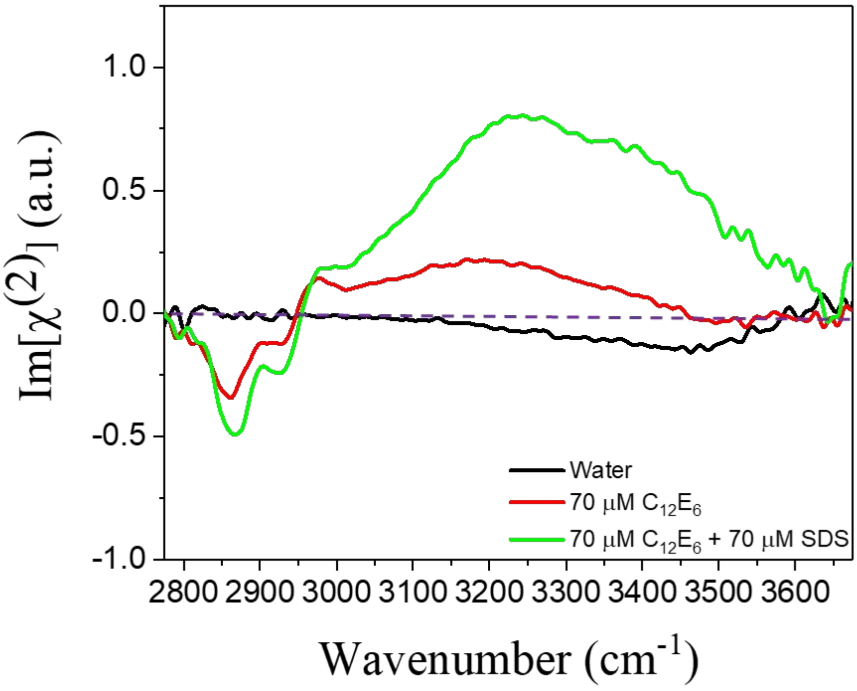

Figure 3: *HDVSFG spectra of pure water (black spectrum), 70 µM $C_{12}E_6$ (red spectrum), and 70 µM $C_{12}E_6$ plus 70 µM SDS (green spectrum) collected at 5 mW infrared power. The spectra don't show any substantial change in the shape and the relative intensity ratio between red and green spectra.*

SI 4: Comparison of decompositions of free energy profiles into enthalpic and entropic components for both systems simulated.

The figures below depict the decomposition of free energy changes calculated from the MD simulation results for both SDS adsorption on the bare-water interface (left) and the water-$C_{12}E_6$ interface (right). In both the panels the blue, green, and orange curves represent the free energy change, potential energy, and +TS for DS^- adsorption process at $C_{12}E_6$ at CMC and bare-water interfaces respectively. The panels show that the free energy change for DS^- adsorption at the $C_{12}E_6$ is much more negative/attractive compared to bare water (-45 k_BT).

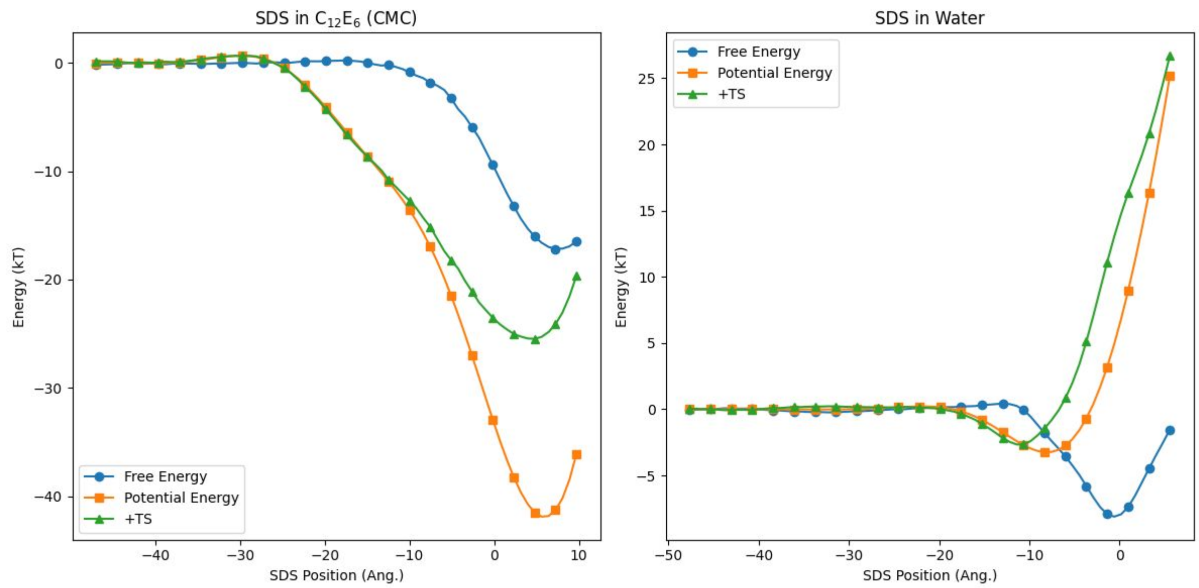

Figure 3: *Comparison between free energy decompositions into enthalpic and entropic components for the DS⁻ ion adsorbing to the air-$C_{12}E_6$-water interface (left) and DS⁻ adsorbing to the bare air-water interface (right).*

SI 5: Energetics breakdown of DS^- adsorption to air-$C_{12}E_6$-water interface vs bare air-water interface.

The two panels show the decompositions of the ΔE due to moving DS^- from the bulk water phase to the air-$C_{12}E_6$-water interface (top panel) and the bare air-water interface (bottom panel). A negative ΔE for a given energy term corresponds to an attractive component of the total system energy that drives DS^- to the interface from the bulk. For the calculations of $\Delta E = E_{\text{int}} - E_{\text{bulk}}$ for each term, the corresponding E_{bulk} was taken as the average potential energy over the range where the free energy is constant, and E_{int} was calculated as an ensemble average over the interfacial region with the (normalized) Boltzmann weight $p(x) \propto \exp[-F(x)/k_B T]$, where $F(x)$ is the free energy along the DS^- position computed with umbrella sampling.

The various decomposition terms are organized into solvent-solvent (vv) and solute-solvent (uv) components, with water, $C_{12}E_6$, and $Na^{(+)}$ corresponding to the solvent, and DS^- corresponding to the solute. The sum of the solvent-solvent and the sum of the solute-solvent components are each shown as well. The black bars correspond to the total system ΔE, representing the total change in enthalpy as a result of DS^- adsorption to each interface.

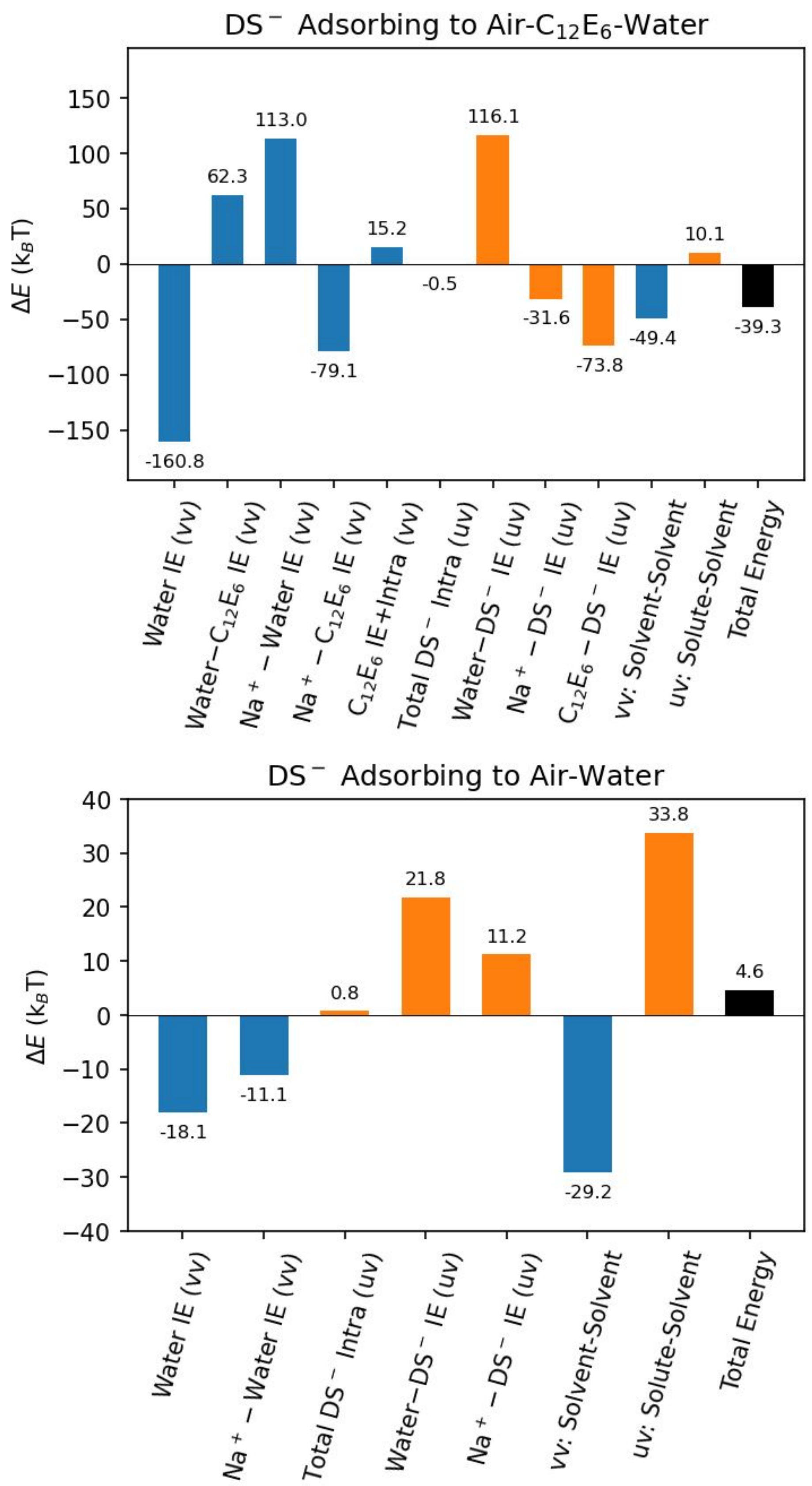

Figure 4: The top panel shows the decompositions for the total energy of DS^- adsorption to the air-$C_{12}E_6$-water interface and the bottom panel depicts the same for the bare air-water interface. The resultant value for the total energy (black bars) for the first process is -39.3 k_BT which is more negative or attractive compared to the later adsorption process is +4.6 k_BT

SI 6: Raw average potential energy data illustrating the averaging method.

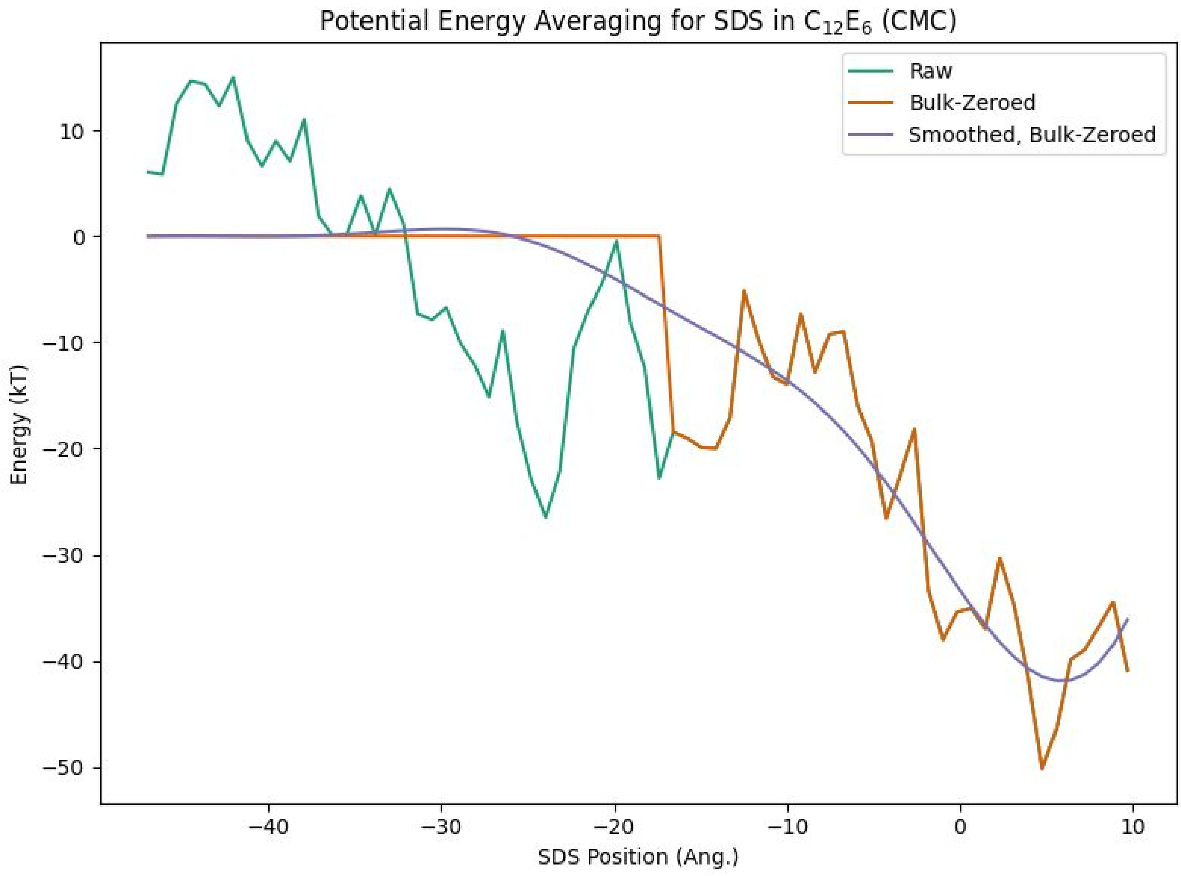

Figure 5: *Average total system potential energy as a function of DS^- ion position relative to the Gibbs dividing interface for DS^- adsorbing to the air-$C_{12}E_6$-water interface. The green curve shows the raw data (averaged into 70 bins), and the orange curve shows the raw data with the bulk values zeroed to the average bulk value (i.e. the potential energy is averaged over all windows in which the free energy is flat), and the purple curve shows the smoothed "Bulk-Zeroed" data using the Savitzky-Golay filter with 3rd-degree polynomials and a window length of 1.31 nm.*

SI 7. Surface tension measurements of the binary mixture

Solutions of $C_{12}E_6$ and SDS in demineralized water (by Mieuxa) were prepared at different concentrations and stirred for an hour. The solutions were used in the 12 hours following their preparation. The interfacial properties at the air/water interface were then probed using an automatic drop tensiometer (Tracker by Teclis). This technique, known as "the pendant drop" method, is based on axisymmetric drop shape analysis and consists in creating a droplet or a rising gas bubble in a solution, extracting the profile of the droplet/bubble using a camera, and

fitting the theoretical Young-Laplace equation to the experimental droplet/bubble profile to extract the value of the surface tension.

The experiments consisted in generating a rising air bubble in a $C_{12}E_6$-SDS solution at a given concentration and monitoring the value of the surface tension at the air/water interface for one hour. Experiments are replicated twice (both at room temperature between 20 and 25 degrees Celcius) and the standard deviations are indicated for each point. The results clearly show that the equilibrium surface tension doesn't vary much at $C_{12}E_6$ excess conditions when for all the cases the concentration of $C_{12}E_6$ was kept at 70 µM and the SDS concentration was varied, i.e. at 1:1 molar ratio both concentrations are 70 µM and at 100:1 ratio the concentration of SDS is 700 nM and $C_{12}E_6$ is at 70 µM.

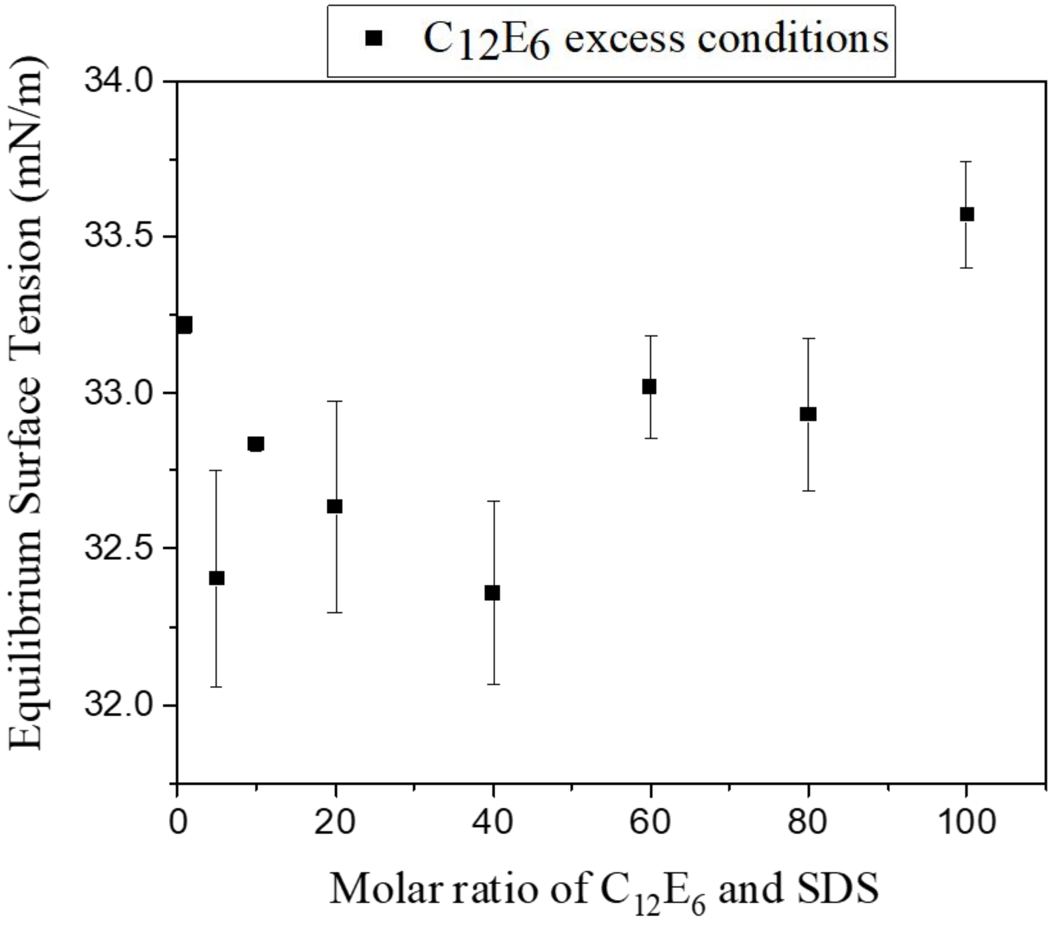

Figure 6: *This figure shows the equilibrium surface tension measurements at $C_{12}E_6$ excess conditions at different molar ratios between $C_{12}E_6$ and SDS. The ratios used here are 1:1, 10:1, 20:1, 40:1, 60:1, 80:1, 100:1.*

SI 8: Materials and Methods:

Materials and preparation of solutions:

A 25 % (wt/vol) solution of $C_{12}E_6$ was brought from Anatrace USA (purity ≥ 99 %) and was diluted to 500 µM solution to prepare further required dilutions using Milipure water (18.2 MΩ-cm). SDS (purity ≥ 99.0 %) was purchased from Sigma. A 10 mM solution was first prepared using an in-house high-precision balance and dissolved in Milipure water. The lower

concentrated solutions were prepared by serial dilution using a solution of 10 mM as the mother solution.

HDVSFG Technique and spectrometer:
Heterodyne-Detected Sum-Frequency Generation spectroscopy (HDVSFG) is an advanced form of vibrational Sum-Frequency Generation Spectroscopy[1-4] (VSFG), allowing the real and imaginary part of the second-order susceptibility associated with surface-specific vibrations. The details of the experimental implementation and the underlying theory of the HDVSFG technique can be found in Refs.[5, 6]. In brief, a mid-infrared (mid-IR) pulse with frequencies ω_{IR} that are in resonance with vibrations of the molecules adsorbed at the surface and a visible pulse (ω_{VIS}) overlap in time and space to generate light at the sum frequency ω_{SFG} of the IR and visible frequencies, where $\omega_{SFG} = \omega_{IR} + \omega_{VIS}$. VSFG is a second-order non-linear process and thus only non-zero becomes non-zero in non-centrosymmetric media within the electric dipole approximation. For random and centrosymmetric media the second-order susceptibility is only non-zero at the surface, where the symmetry is broken. The SFG electric field can be represented as $E_{SFG} \propto \chi^{(2)}\, E_{VIS}\, E_{IR}$ where $\chi^{(2)}$ is the second-ordered non-linear susceptibility and the E terms represent the electric fields of the infrared and visible pulses. The frequency dependence of $\chi^{(2)}$ on the frequency ω_{IR} can be expressed as follows:

$$\chi^{(2)}\,(\,\omega_{SFG} = \omega_{IR} + \omega_{VIS}\,) = A_{NR} + \sum_{i=0}^{n} \frac{A_n}{\omega_n - \omega_{IR} - i\Gamma_n}$$

where ω_n, A_n, and Γ_n are the frequency, amplitude, and damping constant of the nth vibrational mode and A_{NR} is the non-resonant background. In HDVSFG the electric field is phase-sensitively detected by interfering with the field at ω_{SFG} generated by the sample with that of a local oscillator. The direct measurement of the generated electric field avoids interference effects with the non-resonant effect that usually occurs in conventional SFG in which the intensity of the generated light at ω_{SFG} is measured. An additional advantage of the phase-resolved technique over conventional intensity measurements is that it provides direct information on the orientation of the transition dipole moments of the vibrations at the surface and thereby on the orientation of the molecular groups carrying the vibrations.

All measurements were performed with a homebuilt HDVSFG spectrometer[7, 8]. A regenerative Ti: sapphire amplifier (Coherent legend) generates 800 nm (3 mJ, 35 fs) pulses at a repetition rate of 1 kHz. The pulses are divided into two parts to generate the mid-IR and the visible pulses, respectively. A part of 2 mJ is sent to a tuneable homebuilt optical parametric amplifier (OPA) and difference-frequency generator stage. The generated IR pulses have a frequency spectrum centered at 3000 cm^{-1} and an energy of 8 μJ per pulse sent to the sample. The other part of 1 mJ of the fundamental beam is used to generate the visible beam (800 nm) using an etalon. In the HDVSFG technique, we collect VSFG light from both the sample and a local oscillator (gold). The ω_{SFG} beam of the local oscillator is delayed with respect to the ω_{SFG} beam of the sample by passing the local oscillator ω_{SFG} beam through a 1 mm thick silica plate. After passing the two ω_{SFG} beams through a monochromator, the interference pattern of the two beams is collected with an electron-multiplied charged–coupled device (EMCCD- Andor Technologies). Using Fourier transformation, filtering, and back Fourier transformation, both the imaginary and the real parts of the sample HDVSFG electric field are obtained. This electric field is divided by the HDVSFG electric field generated by a reference z-cut quartz crystal, to correct the spectra for the spectral intensity profile of the infrared pulse. All spectra presented in this article are an average of three spectra, typically collected for 60 seconds under constant

nitrogen flow. All presented data are measured with an SSP polarization configuration, where the indexing order of the beams is SFG, visible, and IR.

Computational methods:
Two systems were prepared to model the adsorption process of SDS binding to the air-$C_{12}E_6$ interface, and the bare air-water interface. The former system contains 8038 water molecules and approximately 45 $C_{12}E_6$ molecules on each side. 1 DS^- ion, and 1 Na^+ ion. The initial system consisting of only the $C_{12}E_6$ and water molecules was first run using constant surface tension simulations[9] using the experimental surface tension corresponding to the CMC. This yielded an interfacial surface coverage of 60 molecules per Å^2 which is consistent with previous results[10, 11] The system without the $C_{12}E_6$ contains 8041 water molecules, 1 DS^- ion, and 1 Na^+ ion. Both systems were simulated at constant volume in an orthorhombic box with dimensions 5 nm by 5 nm by 30 nm with periodic boundary conditions in each direction. Simulated at room temperature, the liquid water phase occupies around 10 nm in the z-direction, with the remaining ~ 20 nm representing the vacuum phase.

The US method[12] was used to compute the free energy profile, $G(z)$, of SDS binding to the interface in each system, with z being the distance between the center of mass of DS^- and a weighted[13] center of mass of the liquid water phase projected along the Z-axis. Harmonic bias (umbrella) potentials were used, having the functional form: $w(z) = k/2\ (z - z_0)^2$. In the air-$C_{12}E_6$ system, US windows were placed in 0.1 nm intervals from $z_0 =$ 3.5 nm to 5.7 nm, with (harmonic) spring constants of $k =$ 850 (kJ/mol)/nm. Additional windows for the bulk region were placed at $z_0 =$ 2.7, 3.0, and 3.2 nm with $k =$ 85 (kJ/mol)/nm, at $z_0 =$ 1.8 and 2.3 nm with $k =$ 45 (kJ/mol)/nm, and at $z_0 =$ 0.0 and 1.2 nm with $k =$ 10 (kJ/mol)/nm. In the air-water system, US windows were placed in 0.2 nm intervals from $z_0 =$ 1.5 to 5.3 nm, with $k =$ 320 (kJ/mol)/nm. Additional windows for the bulk region were placed at $z_0 =$ 0.0, 0.8, and 1.2 nm with $k =$ 10 (kJ/mol)/nm. The free energy profiles were unbiased with the weighted histogram analysis method (WHAM)[14]. To decompose the free energy from WHAM into enthalpic and entropic components, the total system potential energy was taken to be a proxy for the enthalpy due to a negligible pV contribution, and the entropic component was taken as the difference from subtracting the enthalpy from the free energy. All energy profiles in Fig. 4 were translated so that $z =$ 0 corresponds to the Gibbs dividing interface defined by the position at which the water density is half of its bulk value (and $z =$ -4.74 nm corresponds to the center of the water box). To reduce the noise in the enthalpy and entropy contributions, the bulk enthalpy was estimated as the average value from $z =$ -4.74 to $z =$ -1.74 (where the free energy is constant). The enthalpy profile within this 3 nm interval was fixed to this value, and the resulting curve was smoothed with 30 passes through the Savitzky-Golay filter[15] with 3rd-degree polynomials and a window length of 15 (corresponding to a window length of 1.31 nm). The raw data is shown in SI 6.

All MD simulations were performed using the open-source package GROMACS[16-22]. All bonds were constrained using the LINCS algorithm[19]. The SPC/E model[23] was employed to describe intermolecular interactions between water molecules, while intramolecular and intermolecular interactions involving SDS and $C_{12}E_6$ were modeled using a combination of the TRaPPE-UA[24] and OPLS[25, 26] force fields described in Ref[10]. A short-range cut-off of 1.0 nm was employed for both the (shifted) Lennard-Jones and Coulomb interactions. Long-range electrostatics were modeled using the Particle Mesh Ewald-Switch[27, 28] method, and a long-range dispersion correction was used correct both the energy and pressure for the cutoff in the van-der-Waals interaction. A time step of 2 fs was used for the Verlet integrator. All simulations were

performed in the canonical ensemble (NVT) at a fixed temperature at 298 K using the stochastic velocity rescaling of Bussi et. al.[29] with a time constant of 0.1 ps.

We also report the integrated dipole moments per unit volume in some of the systems that are simulated. This was computed by adding the water dipole density in the following way:

$$D(z) = \frac{\sum_{t=1}^{M} \sum_{i=1}^{N \in V_z} \mu_z^t(i)}{M. V_z},$$

were $\mu(i)$ is the dipole moment of a single water molecule, $V_z = L_x \times L_y \times dz$, and dz is a small differential in the direction perpendicular to the interface (the z-direction), and L_x, L_y are the sizes of the two remaining dimensions in the simulation box. The variable N corresponds to the number of waters in each volume V_z and M indicates the total number of frames in our simulation.

References:

1) Jubb, A. M.; Hua, W.; Allen, H. C., Environmental Chemistry at Vapor/Water Interfaces: Insights from Vibrational Sum Frequency Generation Spectroscopy. *Annu. Rev. Phys. Chem.* **2012,** *63* (1), 107-130.

(2) Nihonyanagi, S.; Yamaguchi, S.; Tahara, T., Ultrafast Dynamics at Water Interfaces Studied by Vibrational Sum Frequency Generation Spectroscopy. *Chem. Rev.* **2017,** *117* (16), 10665-10693.

(3) Eisenthal, K. B., Liquid interfaces. *Acc. Chem. Res.* **1993,** *26* (12), 636-643.

(4) Wang, H. F.; Velarde, L.; Gan, W.; Fu, L., Quantitative sum-frequency generation vibrational spectroscopy of molecular surfaces and interfaces: lineshape, polarization, and orientation. *Annu. Rev. Phys. Chem.* **2015,** *66*, 189-216.

(5) Shen, Y. R., Phase-Sensitive Sum-Frequency Spectroscopy. *Annu. Rev. Phys. Chem.* **2013,** *64* (1), 129-150.

(6) Nihonyanagi, S.; Ishiyama, T.; Lee, T.-k.; Yamaguchi, S.; Bonn, M.; Morita, A.; Tahara, T., Unified Molecular View of the Air/Water Interface Based on Experimental and Theoretical χ(2) Spectra of an Isotopically Diluted Water Surface. *J. Am. Chem. Soc.* **2011,** *133* (42), 16875-16880.

(7) Strazdaite, S.; Versluis, J.; Bakker, H. J., Water orientation at hydrophobic interfaces. *J. Chem. Phys.* **2015,** *143* (8), 084708.

(8) Moll, C. J.; Giubertoni, G.; van Buren, L.; Versluis, J.; Koenderink, G. H.; Bakker, H. J., Molecular Structure and Surface Accumulation Dynamics of Hyaluronan at the Water–Air Interface. *Macromolecules* **2021,** *54* (18), 8655-8663.

(9) Aoki, K. M.; Yoneya, M.; Yokoyama, H., Constant surface-tension molecular-dynamics simulation methods for anisotropic systems. *J. Chem. Phys.* **2006,** *124* (6), 064705.

(10) Shi, L.; Tummala, N. R.; Striolo, A., C12E6 and SDS Surfactants Simulated at the Vacuum−Water Interface. *Langmuir* **2010,** *26* (8), 5462-5474.

(11) Falciani, G.; Franklin, R.; Cagna, A.; Sen, I.; Hassanali, A.; Chiavazzo, E., A multi-scale perspective of gas transport through soap-film membranes. *Mol. Syst. Des. Eng.* **2020,** *5* (5), 911-921.

(12) Torrie, G. M.; Valleau, J. P., Nonphysical sampling distributions in Monte Carlo free-energy estimation: Umbrella sampling. *J. Comput. Phys.* **1977,** *23* (2), 187-199.

(13) Engin, O.; Villa, A.; Sayar, M.; Hess, B., Driving Forces for Adsorption of Amphiphilic Peptides to the Air− Water Interface. *J. Phys. Chem. B* **2010,** *114* (34), 11093-11101.

(14) Kumar, S.; Rosenberg, J. M.; Bouzida, D.; Swendsen, R. H.; Kollman, P. A., The weighted histogram analysis method for free-energy calculations on biomolecules. I. The method. *J. Comput. Chem.* **1992,** *13* (8), 1011-1021.

(15) Savitzky, A.; Golay, M. J., Smoothing and differentiation of data by simplified least squares procedures. *Anal. Chem.* **1964,** *36* (8), 1627-1639.
(16) Berendsen, H. J.; van der Spoel, D.; van Drunen, R., GROMACS: A message-passing parallel molecular dynamics implementation. *Comput. Phys. Commun.* **1995,** *91* (1-3), 43-56.
(17) Lindahl, E.; Hess, B.; Van Der Spoel, D., GROMACS 3.0: a package for molecular simulation and trajectory analysis. *J. Mol model* **2001,** *7* (8), 306-317.
(18) Van Der Spoel, D.; Lindahl, E.; Hess, B.; Groenhof, G.; Mark, A. E.; Berendsen, H. J., GROMACS: fast, flexible, and free. *J. Comput. Chem.* **2005,** *26* (16), 1701-1718.
(19) Hess, B.; Kutzner, C.; Van Der Spoel, D.; Lindahl, E., GROMACS 4: algorithms for highly efficient, load-balanced, and scalable molecular simulation. *J. Chem. Theory Comput.* **2008,** *4* (3), 435-447.
(20) Pronk, S.; Páll, S.; Schulz, R.; Larsson, P.; Bjelkmar, P.; Apostolov, R.; Shirts, M. R.; Smith, J. C.; Kasson, P. M.; Van Der Spoel, D., GROMACS 4.5: a high-throughput and highly parallel open source molecular simulation toolkit. *Bioinformatics* **2013,** *29* (7), 845-854.
(21) Páll, S.; Abraham, M. J.; Kutzner, C.; Hess, B.; Lindahl, E. In *Tackling exascale software challenges in molecular dynamics simulations with GROMACS*, International conference on exascale applications and software, Springer: **2014**; pp 3-27.
(22) Abraham, M. J.; Murtola, T.; Schulz, R.; Páll, S.; Smith, J. C.; Hess, B.; Lindahl, E., GROMACS: High performance molecular simulations through multi-level parallelism from laptops to supercomputers. *SoftwareX* **2015,** *1*, 19-25.
(23) Berendsen, H.; Grigera, J.; Straatsma, T., The missing term in effective pair potentials. *J. Phys. Chem.* **1987,** *91* (24), 6269-6271.
(24) Martin, M. G.; Siepmann, J. I., Novel configurational-bias Monte Carlo method for branched molecules. Transferable potentials for phase equilibria. 2. United-atom description of branched alkanes. *J. Phys. Chem. B* **1999,** *103* (21), 4508-4517.
(25) Jorgensen, W. L., Optimized intermolecular potential functions for liquid alcohols. *J. Phys. Chem.* **1986,** *90* (7), 1276-1284.
(26) Briggs, J. M.; Matsui, T.; Jorgensen, W. L., Monte Carlo simulations of liquid alkyl ethers with the OPLS potential functions. *J. Comput. Chem.* **1990,** *11* (8), 958-971.
(27) Darden, T.; York, D.; Pedersen, L., Particle mesh Ewald: An N· log (N) method for Ewald sums in large systems. *J. Chem. Phys.* **1993,** *98* (12), 10089-10092.
(28) Essmann, U.; Perera, L.; Berkowitz, M. L.; Darden, T.; Lee, H.; Pedersen, L. G., A smooth particle mesh Ewald method. *J. Chem. Phys.* **1995,** *103* (19), 8577-8593.
(29) Bussi, G.; Donadio, D.; Parrinello, M., Canonical sampling through velocity rescaling. *J. Chem. Phys.* **2007,** *126* (1), 014101.
