# Supplementary material for: Observation of Strong Synergy in the Interfacial Water Response of Binary Ionic and Nonionic Surfactant Mixtures
Source: J Phys Chem Lett. 2022 Dec 1;13(49):11391–7. doi: 10.1021/acs.jpclett.2c02750 (PMC9761666; doi:10.1021/acs.jpclett.2c02750)
Supplement: Supplementary file 2 — jz2c02750_si_002.pdf [file jz2c02750_si_002.pdf]

jz-2022-02750k.R1

Name: Peer Review Information for "Observation of Strong Synergy in the Interfacial Water Response of Binary Ionic and Non-ionic Surfactant Mixtures"

First Round of Reviewer Comments

Reviewer: 1

Comments to the Author

Sengupta et al. investigated surfactant-adsorbed aqueous interfaces using HD VSFG spectroscopy with MD simulation. They found that neutral hexaethylene glycol monododecyl ether (C12E6) adsorbed at the water surface greatly attracts the negatively charged sodium dodecyl sulfate (DS-) when SDS concentration is low, through significant enhancement of the positive OH stretch band in the HD VSFG spectra. Based on the MD simulation, they concluded that this phenomenon arises from the interaction between C12E6 and SDS, which greatly stabilizes the free energy of DS- ion at the water surface due to the change in enthalpy.

In the simple Langmuir adsorption model, adsorbates behave as an ideal gas at isothermal conditions. This study showed that this is not the case for the mixture of even prototypical ionic (SDS) and non-ionic (C16E6) surfactants. The interaction of the two surfactants makes C16E6 attract SDS to the water surface greatly, which was beautifully monitored by HD VSFG as a significant increase of the oriented water reflecting the increase of the surface charge density. The data are clear, the simulation provides consistent results, and their arguments are straightforward. I recommend the publication of this paper in J. Phys. Chem. Lett. However, I suggest several revisions before acceptance. My suggestions are basically minor, but I think that the relevant revisions will improve the readability of this paper. In the following, I list my suggestions in the order of the appearance in the text, not that of importance.

1.

I strongly encourage the authors to show the chemical structures of SDS and C12E6 in the paper, probably in Figure 1.

2.

At the end of the introduction, the authors write, “ … cannot be described with conventional Langmuir adsorption model, “ but they do not explain this statement in the result & discussion part. I understand the meaning, but I am not sure if the meaning is clear also to other physical chemists who are unfamiliar with interface problems. Therefore, I suggest explicitly explaining what the authors want to say with this statement in the last part of this paper, with a brief description of the Langmuir adsorption model.

3.

In the 2nd paragraph on page. 4, there are some inconsistencies in the number of the peak frequencies of the bands, e.g., 2960 vs. 2965 and 2920 vs. 2930.

4.

They show the HD VSFG spectra of SDS solutions in Figure S1, but the spectra in the CH stretch region are missing. Because the alignment of DS- is reflected in this region and it is an essential issue in this study, it is necessary to also show the spectra in the CH region for a fair comparison.

5.

The MD simulation clearly indicates that the observed effect arises from the significant interaction between DS- and C12E6. I want the authors to add some descriptions about the nature of the interaction, e.g., what type of interaction is important, which moieties of the two surfactants are essential for the interaction, etc.

Reviewer: 2

Comments to the Author

Review of "HDVSFG of surface mixture on water.."

this paper present the effect of a small amount of SDS to the C12E6 surfactant on the water surface.

this paper is not recommended due to several important discrepancies noted below.

1. the influence on the water signal could be due to the change orientation, narrower orientation distribution, or a thicker oriented layer. There is no experiential data to help support this, only MD which is not sufficient to make this claim. more polarization data could help verify which effect is important, see Tyrode et al.

2. SDS is not detected, use of d-SDS or d-C12E6 would make this more convincing. The SI also does not show this spectrum which is deficit to the presentation

3. in surfactant studies such as this surface tension is needed to support the conclusion, not provided. How can the authors claim such a violation of Langmuir isotherms when the basic input data is not collected?

4. important work on this system by Richmond, Tyrode, and Bain is not shown, if it was, the authors would see their work is not consistent with the earlier studies

5. assignment in CH region do not look correct.

CH2 sym is at 2850 cm-1 and it FR at 2920. See HF Wang

the CH3 asym. is opposite phase of the the other resonance

the poor resolution makes assignments here unreliable and likely obscures other features in the CH region

full discussion of water peaks should be included such as the negative band at 3450cm-1 for C12E6 but goes positive for SDS addition. further the conc dependent C12E6 should be included

the description of water spectrum has several possibilities and the authors need to present some context to this

6. side note, why the the TOC show a UV beam for the input when the SI says its 800nm?

Author's Response to Peer Review Comments:

Dear Professor Editor,

We are glad to hear your response on our Manuscript, titled 'Observation of Strong Synergy in the Interfacial Water Response of Binary ionic and non-ionic Surfactant Mixtures'.

We have now prepared the reviewers comments and their reply on a point-to point fashion where we have addressed the questions/comments/ suggestions raised by both the reviewers. We have attached files that contaions reviewers' comments replies, updated manuscript and SI marked with yellow coloured texts that we have added/edited. We have also attached the clean manuscript and SI with no colored or highlighted texts for publications. We have also attached the updated TOC, the updated figure 1 and ofcourse, have edited all the references (both in manuscript and SI) as per the JPCL requirements.

We hope these documents are suffiecient for the requested revision and we are looking forward to hear a fruitful outcome of this manuscript. If you have any further questions, please contact me at s.semgupta@amolf.nl.

On behalf of all the authors, we thank for your time and consideration.

Best,

Sanghamitra.

Observation of Strong Synergy in the Interfacial Water Response of Binary ionic and non-ionic Surfactant Mixtures

Reviewers' comments reply

Reviewer 1

We thank the reviewer for the comments and suggestions. Below we answer the comments in the order in which they appeared in the report.

Reviewer comment: I strongly encourage the authors to show the chemical structures of SDS and $C_{12}E_6$ in the paper, probably in Figure 1.

Author reply: *We thank the reviewer for this suggestion. We now include the chemical structures of SDS and $C_{12}E_6$ in Figure 1 as illustrated below. The new figure is implemented in the revised manuscript and the figure caption is updated.*

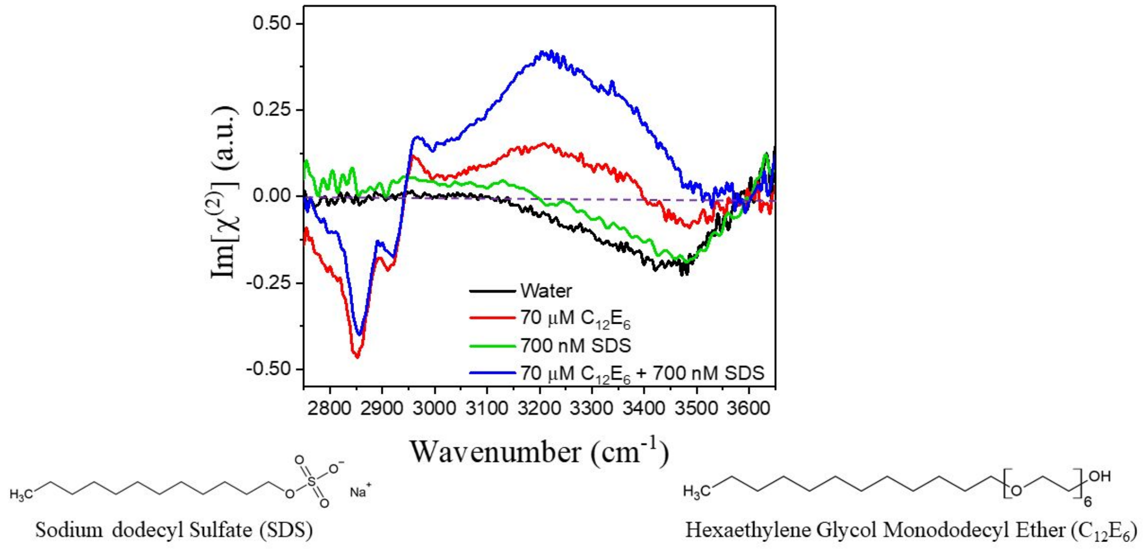

Reviewers comment: At the end of the introduction, the authors write, " … cannot be described with conventional Langmuir adsorption model, " but they do not explain this statement in the result & discussion part. I understand the meaning, but I am not sure if the meaning is clear also to other physical chemists who are unfamiliar with interface problems. Therefore, I suggest explicitly explaining what the authors want to say with this statement in the last part of this paper, with a brief description of the Langmuir adsorption model.

Author reply: *We thank the reviewer for this suggestion. The mathematical expression of the Langmuir model for binary mixture is now added to the supplementary information. We also added the following sentence and paragraph to the manuscript (marked in yellow):*

On page 3:

"The Langmuir adsorption model for both single solute systems and binary mixtures is presented and discussed in SI 1"

On the last paragraph of page 8:

"According to the Langmuir model isotherm (mathematical formula given in SI 1), surfactants will compete for the available surface area. As a result, the surface occupancy of a surfactant will always become lower when another surfactant is added to the solution. As the surface signal of a particular surfactant is proportional to its surface occupancy, the signal collected from the surface of a mixture should be lower than the sum of the signals coming from the surfaces of solutions containing the separate components. Here we observe the opposite. The HDVSFG signal from the binary mixture is strongly enhanced compared to the individual components' surface signals, which shows a clear violation of the Langmuir adsorption isotherm model".

Reviewer's comment: In the 2nd paragraph on the page. 4, there are some inconsistencies in the number of the peak frequencies of the bands, e.g., 2960 vs. 2965 and 2920 vs. 2930.

Author reply: *The authors thank the reviewer for pointing this out. All the frequencies are now consistent throughout the text (highlighted in yellow in the text of the revised manuscript).*

Reviewer's Comment: They show the HD VSFG spectra of SDS solutions in Figure S1, but the spectra in the CH stretch region are missing. Because the alignment of DS- is reflected in this region and is an essential issue in this study, it is necessary to also show the spectra in the CH region for a fair comparison.

Author reply: *We thank the reviewer for this comment. Figure 1 in the SI reporting the HDVSFG spectra of different SDS solutions in pure water, is now extended to a lower frequency of 2800 cm^{-1} which comprises the frequencies of the C-H stretch vibrations. We also added a new figure to the SI (Figure S2) showing the signal measured for the maximum bulk SDS concentration used in this manuscript (70 µM) and higher SDS concentrations. These figures show that the C-H stretch vibrations are only observed when the bulk SDS concentration is >350 µM. We conclude that at the maximum bulk SDS concentration in water of 70 µM, used in this manuscript, the surface density and the alignment of the DS^- ions are not sufficient to produce a clear C-H response.*

<u>Reviewer's Comment:</u> The MD simulation clearly indicates that the observed effect arises from the significant interaction between DS- and $C_{12}E_6$. I want the authors to add some descriptions about the nature of the interaction, e.g., what type of interaction is important, which moieties of the two surfactants are essential for the interaction, etc.

Author reply: *We thank the reviewer for the comment and the suggestion. We have included a description of the type of interaction at the bottom of the first paragraph of page 7, marked in yellow:*

"From our simulations, we find that the large enthalpic stabilization is driven by a combination of van der Waals packing interactions between the hydrophobic chains (~80%) and electrostatic interactions of the ether groups of the head group of $C_{12}E_6$ with the head group of DS (~20%)."

Reviewer 2

The authors thank the reviewer for the comments and suggestions. Below we answer the comments in the order in which they appeared in the report.

Reviewer's Comment: the influence on the water signal could be due to the change orientation, narrower orientation distribution, or a thicker oriented layer. There is no experiential data to help support this, only MD which is not sufficient to make this claim. more polarization data could help verify which effect is important, see Tyrode et al.

Author reply: *We respectfully disagree with the reviewer that there would be no experimental evidence of enhanced water orientation in our manuscript. We observe that the water signal observed in HDVSFG spectra increases when we increase the concentration of a surfactant with a charged head group, like DS^- (in the low concentration regime). This increase in the water signal is due to the enhanced orientation of water molecules in the solution layers near the surface). This enhanced orientation can result from a narrower orientational distribution in the water layer closest to the surface, from an enhanced depth over which water molecules are oriented, and most likely from both effects. In our experiment, the distinction between these two effects is not relevant, it is only relevant that both scale with the surface concentration of charged surfactants. In our work, we study the concentration of the charged surfactant DS^- for binary mixtures of SDS and $C_{12}E_6$ of different compositions. Figure 1 shows that the HDVSFG response of surface water is much more increased when adding 700 nM of SDS to a solution that is already covered with $C_{12}E_6$, than when adding the same amount of SDS to pure water. This result shows that the surface concentration of DS^- is strongly enhanced when $C_{12}E_6$ is present at the surface, thus pointing at a strong synergetic effect of the two surfactants. Using MD simulations, we explain this synergetic effect from a favorable enthalpic interaction of the two surfactants.*

Reviewer's comment: SDS is not detected, use of d-SDS or d-C12E6 would make this more convincing. The SI also does not show this spectrum which is deficit to the presentation

Author Reply: *Following point 4 of the report of Reviewer 1 we have extended the frequency scale of the spectra shown in Figure S1 down to 2800 cm^{-1}, to include the responses of the C-H stretch vibrations of the DS^- aliphatic tail. Up to concentrations of 70 μM no significant C-H signal was observed in the spectra. We also included a figure showing HDVSFG spectra measured at concentrations of SDS between 70 and 700 μM (Figure S2). This figure shows that the C-H vibrations start to appear in the spectra at 350 μM SDS concentration.*

Reviewers comment: _in surfactant studies such as this surface tension is needed to support the conclusion, not provided. How can the authors claim such a violation of Langmuir isotherms when the basic input data is not collected?

Author Reply: *Following the suggestion of the reviewer, we have added surface tension data to the manuscript, presented in Figure 7 of the SI. These data do not show strong changes in the surface tension upon adding small amounts of SDS, which shows that subtle changes in surface density like those studied in our manuscript, can be more sensitively detected with HDVSFG.*

We do not agree with the reviewer that a violation of Langmuir isotherm behavior can exclusively be demonstrated with surface tension data. According to the Langmuir model isotherm (mathematical formula given in the SI), surfactants compete for the available surface area. As a result, the surface occupancy of a surfactant will always become lower when another surfactant is added to the solution. As the surface signal of a particular surfactant is proportional to its surface occupancy, the signal collected from the surface of a mixture should be lower than the sum of the signals coming from the surfaces of solutions containing the separate components. Here we observe the exact opposite. The HDVSFG signal from the binary mixture is strongly enhanced compared to the individual components' surface signals, which shows a clear violation of the Langmuir adsorption isotherm model.

We added a paragraph to page 8 explaining that the HDVSFG data provide clear evidence for the violation of the Langmuir isotherm model for binary mixtures of $C_{12}E_6$ *and SDS.*

Reviewer's comment: important work on this system by Richmond, Tyrode, and Bain is not shown, if it was, the authors would see their work is not consistent with the earlier studies

Author Reply: *We are confused by this remark. We have done a thorough search but have not found any journal article on a study with SFG of binary mixtures of SDS and* $C_{12}E_6$ *by the three researchers mentioned by the reviewer. There have been some studies on other combinations of mixed surfactants. Geraldine Richmond has studied binary mixtures of cationic and anionic surfactants (doi.org/10.1021/ja962277y), and Colin Bain has done work on mixed non-ionic surfactants (doi:10.1016/j.cis.2009.12.002). None of these systems resemble the ionic-neutral SDS-*$C_{12}E_6$ *system studied in this manuscript. Moreover, in these other studies, the molar ratio of the studied binary mixtures and the other experimental conditions are in a very different range compared to our work. Possibly the reviewer refers to work on solutions only containing surfactants homologous to* $C_{12}E_6$ *(no SDS), see the next point.*

Reviewer's comment: assignment in CH region do not look correct.
CH2 sym is at 2850 cm-1 and it FR at 2920. See HF Wang
the CH3 asym. is opposite phase of the the other resonance
the poor resolution makes assignments here unreliable and likely obscures other features in the CH region
full discussion of water peaks should be included such as the negative band at 3450cm-1 for $C_{12}E_6$ but goes positive for SDS addition. further the conc dependent C12E6 should be included
the description of water spectrum has several possibilities and the authors need to present some context to this

Author reply: *We thank the reviewer for this comment. The revised the assignment of the C-H stretch vibrational bands to make it fully consistent with earlier work of Eric Tyrode et al. on systems that are highly similar to* $C_{12}E_6$ *(J. Phys. Chem. C* ***2007,*** *111, 11642-11652).*

We revised the paragraph on the assignment of C-H stretch vibrations. The new paragraph can be found on page 4 of the revised manuscript, marked in yellow:

"Following earlier work on systems analogous to $C_{12}E_6$ *by the group of Tyrode (ref 37), we assign the band at 2850* cm^{-1} *to the symmetric C-H stretch vibrations of the methylene (*CH_2*) groups and the terminal* CH_3 *group of the aliphatic chain of* $C_{12}E_6$*, with a dominant contribution of the* CH_2 *groups. The negative band at 2920* cm^{-1} *is assigned to the fermi-resonance of the symmetric C-H stretch vibrations and the overtones of the C-H bending mode of the* CH_2 *and* CH_3 *groups (ref 30,38)".*

We added the origin of the water signal at 3450 cm^{-1} *on page 5 of the revised manuscript, marked in yellow:*

"The positive water response of $C_{12}E_6$ *just by itself is explained in detail elsewhere (ref 29). To briefly mention, the positive water signal between 3000 and 3400* cm^{-1} *for solutions of* $C_{12}E_6$ *results from the strong hydrogen bonding of water molecules to the ether groups of the head group of* $C_{12}E_6$*. The negative water signal around 3450* cm^{-1} *observed for a solution containing only* $C_{12}E_6$ *has been attributed to weakly hydrogen-bonded water molecules located in between the hydrophobic tails of the surfactant molecules. For these water molecules the O-H groups are oriented towards the bulk, thus yielding a negative HDVSFG signal. The addition of SDS generates an overall broad positive water signal due to the strong orientation effect of the negative charge on the water near the surface. This signal overshadows the negative water signal of the water molecules in between the hydrophobic tails of* $C_{12}E_6$*, leading to a net positive signal at all frequencies."*

The dependence of the HDVSFG signal on the concentration of $C_{12}E_6$ *has been published before. We added a citation to this work (reference 29).*

Reviewer's comment: side note, why the TOC show a UV beam for the input when the SI says it's 800nm?

Authors reply: *We thank the reviewer for pointing out this error. We corrected the TOC.*
